# New York College of Veterinary Surgeons—Commencement

**Published:** 1892-04

**Authors:** 


### NEW YORK COLLEGE OF VETERINARY SURGEONS— COMMENCEMENT.

The New York College of Veterinary Surgeons, held its commencement at Chickering Hall, New York, on Friday, March 18th. The diploma was granted to the following gentlemen:

P. J. Bryan, Brooklyn, N. Y.; G. W. Browning, Lincoln, Ill.; R. J. Church, Grand Forks, North Dakota,; A. K. Davidheiser, Pottstown, Pa.; Van C. Dull, Phillipsburg, N. J.; Henry Dutcher, U. S. A., West Point, N. Y.; H. W. Eliot, New Haven, Conn.; V. H. Gibson, Upper Sandusky, O.; D. W. Gilbert, South Salem, N. Y.; S. A. Honeywell, Cleveland, O.; Walter Johnson, More, Pa.; Ferdinand Hueppe, Berlin, Germany; S. B. McDougall, Grove City, Pa.; Wm. McLoughlin, New York City; John McTammany,

Brooklyn, N. Y.; John P. Matthews, Lambertville, Pa.; Enos S. Moyer, Hillstown, Bucks Co., Pa.; A. Peschmanns, Lakegrove, L. I.; Hervey T. Potter, New Haven, Conn.; Benj. Schmidt, New Bremen, O.; Stanley Smith, Columbia, Mo.; Charles Seay, Frankfort, Ky.; Chas. Seilmann, New York City; William Swan, New York City; Eli Straus, New York City; Harrison F. West, New York City; Walter Howard Wilson, Philadelphia, Pa.; Fred Winters, White Lake, N. Y.; Harry S. Willis, Rapidan, Va.

